# Active Design Method for the Static Characteristics of a Piezoelectric Six-Axis Force/Torque Sensor

**DOI:** 10.3390/s140100659

**Published:** 2014-01-02

**Authors:** Jun Liu, Min Li, Lan Qin, Jingcheng Liu

**Affiliations:** 1 Key Laboratory of Optoelectronics Technology and Systems Ministry of Education, Chongqing University, Chongqing 400044, China; E-Mails: limin780815@cqu.edu.cn (M.L.); qinlan@cqu.edu.cn (L.Q.); jcliu@cqu.edu.cn (J.L.); 2 College of Optoelectronic Engineering, Chongqing University, Chongqing 400044, China

**Keywords:** finite element, static characteristic, six-axis force/torque, piezoelectric sensor, active design theory

## Abstract

To address the bottleneck issues of an elastic-style six-axis force/torque sensor (six-axis force sensor), this work proposes a no-elastic piezoelectric six-axis force sensor. The operating principle of the piezoelectric six-axis force sensor is analyzed, and a structural model is constructed. The static-active design theory of the piezoelectric six-axis force sensor is established, including a static analytical/mathematical model and numerical simulation model (finite element model). A piezoelectric six-axis force sensor experimental prototype is developed according to the analytical mathematical model and numerical simulation model, and selected static characteristic parameters (including sensitivity, isotropic degree and cross-coupling) are tested using this model with three approaches. The measured results are in agreement with the analytical results from the static-active design method. Therefore, this study has successfully established a foundation for further research into the piezoelectric multi-axis force sensor and an overall design approach based on static characteristics.

## 1. Introduction

A six-axis force sensor is a device designed for measuring external forces and collecting spatial force information from three force components (Fx, Fy, Fz) and three torque components (Mx, My, Mz). Such a device also detects the position information of the force functional point. These sensors play significant roles in space robot design, space station docking simulations, rocket engine thrust testing, rocket-assisted aerodynamic characteristics testing, the collection of real time center position information for a flexible seating system, machine health monitoring and other applications. According to the GB7665-87 national standard, the six-axis force sensor can be classified as either elastic style [1,2] or non-elastic style [3].

Currently, three bottleneck issues exist in the elastic-style six-axis force sensor, including a degree of structural complexity and difficulty in decoupling [4], high stiffness and high sensitivity [5] and issues of elastic quality and degree of cross coupling [6]; these issues affect its performance, create obstacles to further enhancement, and restrict further expansion of its application space. Therefore, researchers have carried out studies on a non-elastic style six-axis force sensor with component forces acting directly on the sensing elements. This sensor uses piezoelectric components as the sensing element and conversion element, and when combined with a multi-point support structure, this device is able to measure the six-axis forces. However, at present, few research results are available. Didler [7] and Liu [8] developed a Stewart-structure piezoelectric six-axis force sensor that embedded quartz crystal chips into the six legs of the Stewart platform. Li [9] researched a large-range six-axis force sensor based on the six-axis force platform of the Kistler company. The devices in these studies are able to overcome the bottleneck issues of the elastic-style six-axis force sensor. However, these research programs were aimed at a specific goal, and a universal analytical/mathematical model has yet to be developed. As a result, it is difficult to achieve miniaturization of the sensor. For this reason, this work [3] proposes a piezoelectric six-axis force research program based on an eight-point support structure that can deliver miniaturization [10] and enhance the dynamic performance of the sensor. However, due to the influence of the electrode plates, the performance of this device is affected by environmental humidity, and thus its stability must be further strengthened.

The ultimate goal for the static design of the piezoelectric six-axis force sensor is to realize an active design based on its static performance. This effort requires research into the isotropic characteristics that affect the measurement accuracy [11], and more importantly, it requires comprehensive study and design of the device's static performance. The research method uses a high-precision analytical/mathematic model to deliver an optimal design of the piezoelectric six-axis force sensor. Furthermore, this work uses the conclusions of the numerical simulation model to optimize the analytical/mathematical model and verify its effectiveness and uses the experimental results to verify the correctness of analytical model and numerical model (*i.e.*, to build the active design theory for these sensors). Active design theory plays an important role in the static design of a six-axis force sensor, and the static analytical/mathematical model acts as its foundation [12]. Compared with single-axis force sensors, the six-axis force sensor's structure is more complex, and thus it is more difficult to deduce an effective analytical/mathematical model. Due to these difficulties, many different types of six-axis force sensors have been proposed, but no one has been able to identify the in-depth design theory, and therefore, studies of the characteristics of these sensors are still carried out solely by experimental calibration.

To meet the need for a piezoelectric six-axis force sensor active design based on its static characteristics, this work researches the active design method of the piezoelectric six-axis force sensor's static characteristics based on preliminary studies. Piezoelectric quartz is selected for the force sensing elements. The operating principle of the six-axis force sensor is analyzed, and an eight-point support structure based on a double quartz crystal chip group is proposed. Furthermore, a static analytical/mathematical model is built, a numerical finite element model of the piezoelectric six-axis force sensor is set up, and the active design theory of this type of sensor is established. The correctness of the active design theory is verified by the calibration results from the piezoelectric six-axis force sensor experimental prototype, and the conclusions of the study reveal the main factors that affect the six-axis force sensor's static characteristics.

## 2. Measurement Principle and Structure Model

According to the structural characteristics of the piezoelectric element inside the piezoelectric force sensor, the force sensor can be divided into two types, including the integral structure and disaggregated structure. The integral structure piezoelectric force sensor consists of an internal piezoelectric element in the form of a complete wafer or annular plates. The disaggregated structure of the piezoelectric force sensor includes a number of piezoelectric elements, which are evenly arranged according to specific rules. The integral structure can reduce the cross-sectional area of the sensor, but its number of measurement dimensions cannot exceed four, which is not suitable for large-size structures. Therefore, it is difficult to miniaturize this sensor using MEMS technology. Therefore, a piezoelectric element multi-point support structure should be used if the piezoelectric elements are expected to achieve multi-axis force measurements of more than four dimensions.

### 2.1. Measurement Principle

**Figure 1.**
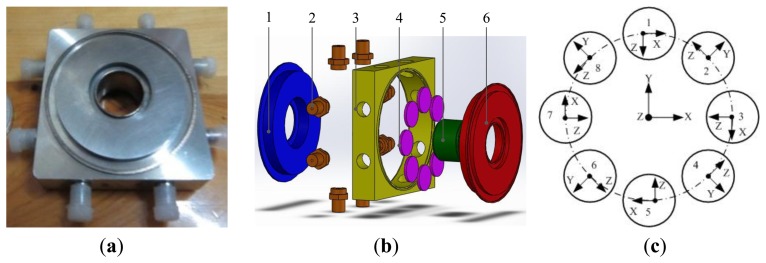
Schematic diagram of piezoelectric 6-axis force sensor. (**a**) Photo of a piezoelectric six-axis force sensor. (**b**) Exploded view of a six-axis force sensor. (**c**) Spatial layout structure schematic of quartz chip groups.

The experimental prototype of the piezoelectric six-axis force sensor is shown in Figure 1a. Piezoelectric quartz is chosen for the sensing element and the conversion element of this six-axis force sensor. Assuming that the measured forces/torques are *f_x_*, *f_y_*, *f_z_*, *m_x_*, *m_y_* and *m_z_*, the piezoelectric quartz crystal chip groups response outputs are *F_X_*, *F_Y_*, *F_Z_*, *M_X_*, *M_Y_* and *M_Z_*. The piezoelectric six-axis force sensor is composed of upper cover (1), electrode (2), shell (3), inner cylinder (4), piezoelectric quartz crystal chip group (5) and lower cover (6). The quartz crystal chip groups are clamped between the upper cover and lower cover and are symmetrically distributed on the upper surface of the quartz crystal chip group's mounting boss. To improve the impedance characteristics and anti-jamming performance of the piezoelectric six-axis force sensor (as mentioned in reference [1]), the piezoelectric quartz crystal chip groups are designed for a double-layer structure.

Figure 1b shows the structural schematic and layout of the quartz crystal chip groups within the piezoelectric six-axis force sensor. Eight quartz crystal chip groups are evenly distributed on the same distribution circle. Four groups of Y0^0^-crystals are distributed on the nodes of the X and Y axes and the quartz crystal chip groups distribution circle and are used for the measurement of *f_x_*, *f_y_* and *m_z_*. Four groups of X0^0^-crystals are distributed on the other locations and are used for the measurement of *f_z_*, *m_x_* and *m_y_*. Each group quartz crystal chip group corresponds to a one-channel output signal and can obtain 6-channel signals via pretreatment of 8-channel signals, and the six-axis forces can be measured via operation of a decoupling matrix. Equation (1) represents the 8-channel signal to 6-channel signal conversion expression in which the subscript letters represent the spatial axes and the value index indicates the number of quartz chip groups:

(1) $$\left\{ \begin{array}{l} F_{X}=k_{f_{x}}\left(F_{Y1}-F_{Y5}\right)\\ F_{Y}=k_{f_{y}}\left(F_{Y3}-F_{Y7}\right)\\ F_{Z}=k_{f_{z}}\left(F_{Z2}+F_{Z4}+F_{Z6}+F_{Z8}\right)\\ M_{X}=k_{m_{x}}[(F_{Z2}+F_{Z8})-(F_{Z4}+F_{Z6})]\\ M_{Y}=k_{m_{y}}[(F_{Z6}+F_{Z8})-(F_{Z2}+F_{Z4})]\\ M_{Z}=k_{m_{z}}\left(F_{X1}+F_{X3}+F_{X5}+F_{X7}\right) \end{array}\right.$$

Due to the influence of the piezoelectric six-axis accelerometer structure, the layout of the quartz chip group, the quantity and production level (among other factors), the arrangement of the quantity of quartz crystal cells and the production level, the actual conditions do not fully meet the above assumption in practice. Therefore, the acceleration transfer coefficients of *k_f_x__*, *k_f_y__*, *k_f_z__*, *k_m_x__*, *k_m_y__* and *k_m_z__* were introduced into this study.

### 2.2. Structure Model

To simplify the analysis, the following assumptions are adopted. The rigidities of the quartz crystal chip groups are identical, with equal sensitivity and symmetric uniform distribution. The cover of the piezoelectric six-axis force sensor is a rigid body with the same stiffness in all directions, equal sensitivity and uniform distribution. The directions of *f_z_*, *m_x_* and *m_y_* are distributed according to the lever principle on the quartz crystal chips, and *f_x_*, *f_y_* and *m_z_* are evenly distributed.

**Figure 2.**
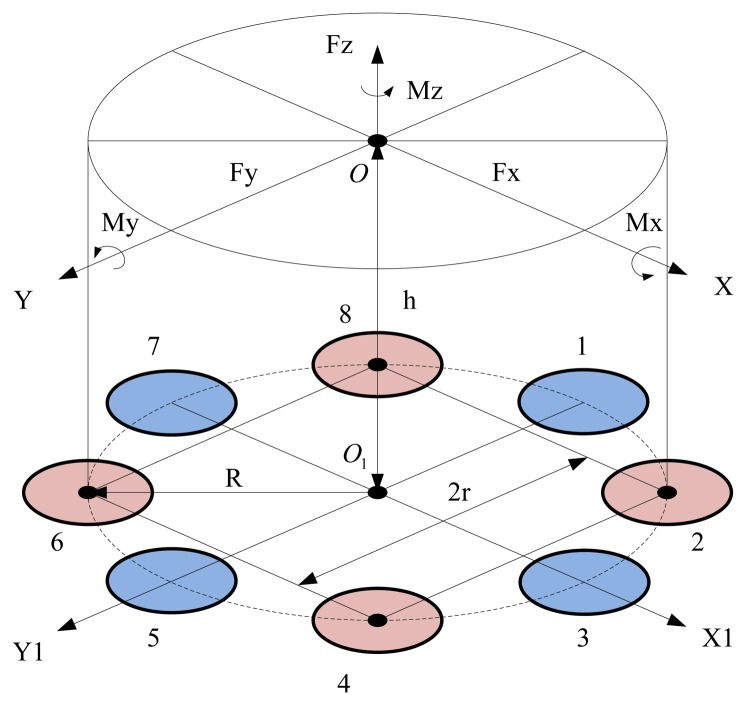
Block diagram of the sensor's structure.

Figure 2 shows the block diagram of the piezoelectric six-axis force sensor structure. The designation O-XYZ represents the coordinate system of the measured force functional point, and O_1_-X_1_Y_1_Z_1_ denotes the installation layout position coordinate system of the quartz crystal chip groups. The quartz crystal chip groups are arranged along the same circle with radius is R, the distance between quartz chip groups 2 and 4 is 2r = 1.414R, and the distance between the force and the surface of quartz crystal chip groups is h. The component forces acting on each quartz crystal chip groups can be expressed by Equation (2):

(2) $$\left\{ \begin{array}{l} F_{X1}=a_{x}/8+am_{z}/8R+am_{y}/R\\ F_{X5}=-a_{x}/8+am_{z}/8R-am_{y}/R\\ F_{Y3}=a_{y}/8+am_{z}/8R-am_{x}/R\\ F_{Y7}=-a_{y}/8+am_{z}/8R+am_{x}/R\\ F_{Z2}=a_{z}/8+[-a_{y}h-a_{x}h+am_{x}-am_{y}]/3r\\ F_{Z4}=a_{z}/8+[a_{y}h-a_{x}h-am_{x}-am_{y}]/3r\\ F_{Z6}=a_{z}/8+[a_{y}h+a_{x}h-am_{x}+am_{y}]/3r\\ F_{Z8}=a_{z}/8+[-a_{y}h+a_{x}h+am_{x}+am_{y}]/3r \end{array}\right.$$

According to the Equations (1) and (2), we can obtain the piezoelectric six-axis force sensor's output charge Equation (3) and linear decoupling matrix *C_Qm_*, where S is the cross-sectional area of the quartz chip, S_e_ is the available cross-sectional area of electrode, d_11_ and d_26_ are the piezoelectric moduli of the quartz crystals:

(3) $$\left\{ \begin{array}{l} Q_{F_{X}}=(f_{x}/2)k_{f_{x}}d_{26}S_{e}/S\\ Q_{F_{Y}}=(f_{y}/2)k_{f_{y}}d_{26}S_{e}/S\\ Q_{F_{Z}}=f_{z}k_{f_{z}}d_{11}S_{e}/S\\ Q_{M_{X}}=(8m_{x}/3Sr-8f_{y}h/3Sr)k_{m_{x}}d_{11}S_{e}\\ Q_{M_{Y}}=(8m_{y}/3Sr-8f_{x}h/3Sr)k_{m_{y}}d_{11}S_{e}\\ Q_{M_{Z}}=8m_{z}k_{m_{z}}d_{26}S_{e}/RS) \end{array}\right.$$

(4) $$C_{Qm}=\frac{S_{e}}{S}\left[\begin{array}{cccccc} k_{f_{x}}d_{26}/2 & 0 & 0 & 0 & 8hk_{m_{y}}d_{11}/3r & 0\\ 0 & k_{f_{y}}d_{26}/2 & 0 & -8hk_{m_{x}}d_{11}/3r & 0 & 0\\ 0 & 0 & k_{f_{z}}d_{11} & 0 & 0 & 0\\ 0 & 0 & 0 & 8k_{m_{x}}d_{11}/3r & 0 & 0\\ 0 & 0 & 0 & 0 & 8k_{m_{y}}d_{11}/3r & 0\\ 0 & 0 & 0 & 0 & 0 & 8k_{m_{z}}d_{26}/R \end{array}\right]$$

As can be seen from Equations (3) and (4), due to the influence of the sensor structure, the cross coupling interferences of six-axis force sensor take place in the *f_y_*, *m_x_*, *f_x_*, *m_y_* directions. These interferences are different from traditional nonlinear coupling, and can be eliminated using a mathematical compensation method.

## 3. Static Characteristics Analysis

### 3.1. Analytical Model

In the process of obtaining a high-precision analytical/mathematical model of the piezoelectric six-axis force sensor from the structural model, the key difficulties lie in solving for the load transfer coefficients *k_f_x*, *k_f_y*, *k_f_z*, *k_m_x*, *k_m_y*_and_ *k_m_z*. To obtain the load transfer coefficients, the following computations should be carried out. First, the transmission path of the six-axis force load on the sensor must be analyzed. Next, the equivalent rigidity and equivalent mass of the piezoelectric six-axis force sensor components should be solved. Finally, the load transfer coefficient expressions can be constructed.

#### Analysis of the load transfer path

**Figure 3.**
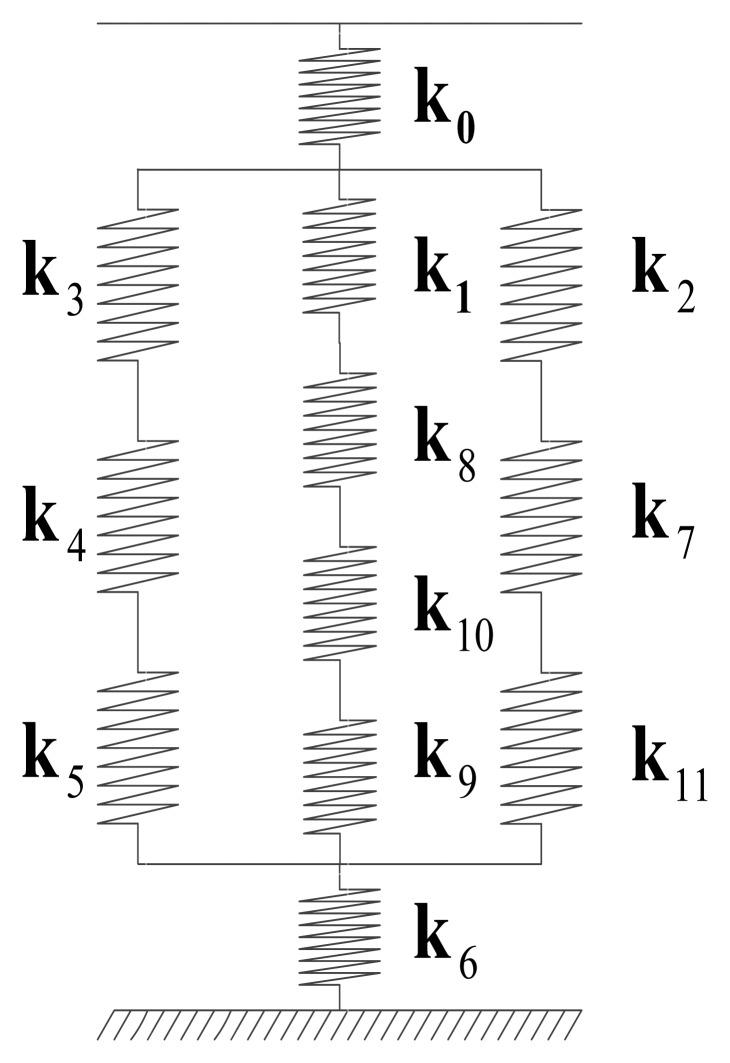
The static spring equivalent model of the piezoelectric six-axis force sensor.

Figure 3 shows the following components: the static spring equivalent model of the piezoelectric six-axis force sensor, where k_0_–k_11_ are the equivalent rigidity of the load functional part of the upper cover; the sensitive part of upper cover; the inner and outer ring elastic modulus of upper cover; the shell, the inner and outer ring elastic modulus of the lower cover; the boss of lower cover; the inner tube; the quartz crystal chip groups, and other components. After the externally measured spatial six-axis force acts on the surface of the upper cover, the force is transferred from the upper boss of the upper cover down to the upper part of the lower cover through the following components: the outer ring, inner the ring elastic modulus and lower boss of the upper cover; the shell; the upper quartz crystal chip groups; the electrode pads; the lower quartz crystal chip groups; the inner tube; the inner and outer ring elastic modulus of lower cover and other components. According to the theory of series and parallel spring's equivalent stiffness [13], the force/torque transfer coefficient of the piezoelectric six-axis force sensor can be expressed by Equation (5). In this expression, the letter subscript (***f*** or ***m***) indicates force or torque and the numerical subscript represents the part number of the piezoelectric six-axis force sensor:

(5) $$k_{f_{x}}/k_{f_{y}}/k_{f_{z}}/k_{m_{x}}/k_{m_{y}}/k_{m_{z}}=\frac{k_{1/8/10/9}}{k_{3/4/5}+k_{1/8/10/9}+k_{2/7/11}}$$

#### Analysis of the equivalent stiffness and equivalent mass

For example, as torque *m_x_* acts on the upper boss surface of the upper cover, the boss of the upper cover, the boss of the lower cover, and a combination of the quartz crystal chip groups receive uniform loads perpendicular to the surface and produce the compression deformation. At the same time, the inner tube and shell experience compression deformation. The inner and outer ring elastic modulus of the upper and lower covers can be considered as a cantilever model without angle of rotation. As discussed previously, according to Equation (5), the composite equivalent stiffness can be obtained from the parameter Equations (6–8). In the parameter expressions, *E_i_* is the Young's modulus, *ρ_i_* is the density, *μ_i_* is Poisson's ratio, *b_i_* is the thickness, *r_i_* is the radius, and *i* (i = 0–11) is the number of piezoelectric six-axis force sensor parts. According to Equations (5–8), the torque *m_x_* transfer coefficient *k_m_x*:

(6) $$\begin{array}{l} k_{1/8/10/9}=\left\{\frac{E_{3}\pi (r_{6}^{2}-r_{5}^{2})}{b_{12}}\frac{E_{1}\pi r_{1}^{2}}{b_{7}}\frac{E_{2}\pi r_{2}^{2}}{b_{8}}\frac{E_{1}\pi r_{1}^{2}}{b_{7}}\right\}/\{\frac{E_{3}\pi (r_{6}^{2}-r_{5}^{2})}{b_{12}}\frac{E_{1}\pi r_{1}^{2}}{b_{7}}\frac{E_{2}\pi r_{2}^{2}}{b_{8}}+\\ \frac{E_{3}\pi (r_{6}^{2}-r_{5}^{2})}{b_{12}}\frac{E_{1}\pi r_{1}^{2}}{b_{7}}\frac{E_{1}\pi r_{1}^{2}}{b_{7}}+\frac{E_{3}\pi (r_{6}^{2}-r_{5}^{2})}{b_{12}}\frac{E_{2}\pi r_{2}^{2}}{b_{8}}\frac{E_{1}\pi r_{1}^{2}}{b_{7}}+\frac{E_{1}\pi r_{1}^{2}}{b_{7}}\frac{E_{2}\pi r_{2}^{2}}{b_{8}}\frac{E_{1}\pi r_{1}^{2}}{b_{7}}\} \end{array}$$

(7) $$\begin{array}{c} k_{3/4/5}=\left\{\frac{E_{3}b_{3}\pi^{3}{\left(r_{7}+r_{6}\right)}^{3}}{{\left(r_{7}-r_{6}\right)}^{3}}\frac{E_{3}\pi }{b_{10}}(r_{8}^{2}-r_{7}^{2})\frac{E_{3}b_{6}\pi^{3}{\left(r_{7}+r_{10}\right)}^{3}}{{\left(r_{7}-r_{10}\right)}^{3}}\right\}/\{\frac{E_{3}b_{3}\pi^{3}{\left(r_{7}+r_{6}\right)}^{3}}{{\left(r_{7}-r_{6}\right)}^{3}}\frac{E_{3}\pi }{b_{10}}(r_{8}^{2}-r_{7}^{2})+\\ \frac{E_{3}b_{3}\pi^{3}{\left(r_{7}+r_{6}\right)}^{3}}{{\left(r_{7}-r_{6}\right)}^{3}}\frac{E_{3}b_{6}\pi^{3}{\left(r_{7}+r_{10}\right)}^{3}}{{\left(r_{7}-r_{10}\right)}^{3}}+\frac{E_{3}\pi }{b_{10}}(r_{8}^{2}-r_{7}^{2})\frac{E_{3}b_{6}\pi^{3}{\left(r_{7}+r_{10}\right)}^{3}}{{\left(r_{7}-r_{10}\right)}^{3}}\} \end{array}$$

(8) $$\begin{array}{c} k_{2/7/11}=\left\{\frac{E_{3}b_{2}\pi^{3}{\left(r_{4}+r_{5}\right)}^{3}}{{\left(r_{5}-r_{4}\right)}^{3}}\frac{E_{3}\pi (r_{4}^{2}-r_{3}^{2})}{b_{9}}\frac{E_{3}b_{5}\pi^{3}{\left(r_{9}+r_{4}\right)}^{3}}{{\left(r_{9}-r_{4}\right)}^{3}}\right\}/\{\frac{E_{3}b_{2}\pi^{3}{\left(r_{4}+r_{5}\right)}^{3}}{{\left(r_{5}-r_{4}\right)}^{3}}\frac{E_{3}\pi (r_{4}^{2}-r_{3}^{2})}{b_{9}}+\\ \frac{E_{3}b_{2}\pi^{3}{\left(r_{4}+r_{5}\right)}^{3}}{{\left(r_{5}-r_{4}\right)}^{3}}\frac{E_{3}b_{5}\pi^{3}{\left(r_{9}+r_{4}\right)}^{3}}{{\left(r_{9}-r_{4}\right)}^{3}}+\frac{E_{3}\pi (r_{4}^{2}-r_{3}^{2})}{b_{9}}\frac{E_{3}b_{5}\pi^{3}{\left(r_{9}+r_{4}\right)}^{3}}{{\left(r_{9}-r_{4}\right)}^{3}}\} \end{array}$$

### 3.2. Numerical Model

To verify the effectiveness of the piezoelectric six-axis force sensor analytical mathematical model, ANSYS software is used to pre-assess the piezoelectric six-axis force sensor static characteristics. The analysis process primarily applies a modeling approach and load application method.

#### Modeling approach

In the first step, the physical structural model of the piezoelectric six-axis force sensor is built with CAD software (*i.e.*, SolidWorks or PRE/E), and the physical structural model is imported into the ANSYS software. The element types and material parameters of the model can be defined according to the material characteristics of the piezoelectric six-axis force sensor components. The piezoelectric six-axis force sensor's working coordinates and the quartz crystal chip groups' local coordinates can be constructed according to the operating conditions of the sensor and the digestion type of the quartz crystal chips. Finally, according to the actual computational requirements, the meshing method is determined to complete the meshing of piezoelectric six-axis force sensor.

#### Load application method

**Figure 4.**
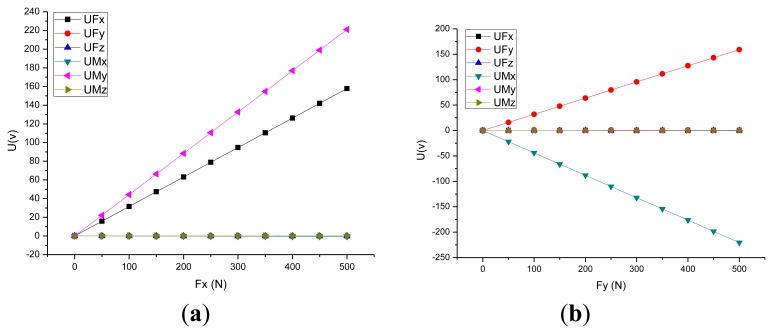

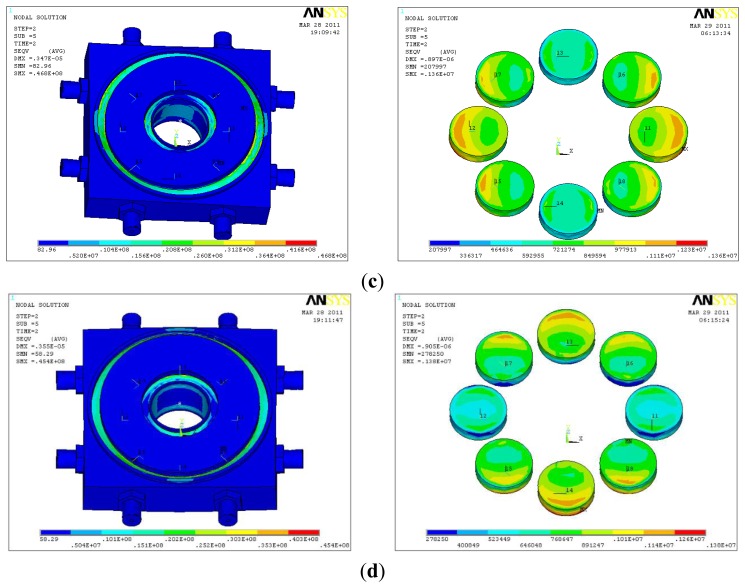
Selected input and output simulation characteristics of the sensor: (**a**) Force-potential curve in the F_X_ direction, (**b**) Force-potential curve in the F_Y_ direction, (**c**) Strain cloud of the sensor and quartz crystal chip groups under F_X_, (**d**) Strain cloud of the sensor and quartz crystal chip groups under F_Y_.

This approach includes the installation constraints and acting force/torque loading on the piezoelectric six-axis force sensor. The constraints set adheres to the sensor's installation status, and the preload force is applied through a section of the cover. The degree of freedom of the pedestal's mounting surface is zero. The measured force/moments are applied to the key point, which is established on the Z-axis and located in the same plane as the upper surface of the piezoelectric six-axis force sensor. Additionally, the key point and the upper surface of the sensor's cover are built in the rigid region. Figure 4a,b show the curves between the input force and the output potential in the X and Y directions. Under *f_x_*(*f_y_*), the strain of quartz chip groups 1,5 (3,7) is most uniform(symmetric).

Figure 4c,d shows the stress contour of the piezoelectric six-axis force sensor finite element model and the quartz crystal chip groups when a horizontal load of *f_x_* = *f_y_* = 500 N is applied. It can be observed that the input load is linear with the output potential and the cross-coupling between *f_x_*(*f_y_*) to *m_y_*(*m_x_*) is linear in the opposite direction. These conclusions are consistent with the analytical/mathematical model of the piezoelectric six-axis force sensor.

## 4. Experimental Results and Discussion

An experimental prototype of the piezoelectric six-axis force sensor was constructed to verify the validity of the piezoelectric six-axis force sensor analytical/mathematical model and numerical simulation model. A conclusion can be obtained by comparing the analytical model, numerical model and experimental calibration results of the piezoelectric six-axis force sensor with the same structure. Table 1 lists the main structural dimensions of the sensor examined in this study.

**Figure 5.**
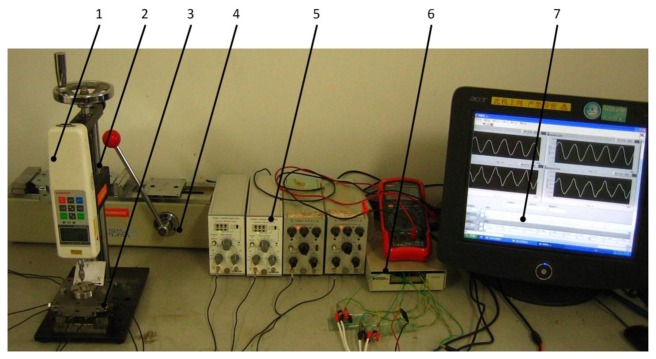
Photo of the static calibration test system: (1) Static standard power source. (2) Vertical loading device, (3) Six-axis force sensor, (4) Transverse loading device, (5) Quasi-static charge amplifier, (6) Pre-processing circuit, (7) Acquisition software.

Figure 5 shows the piezoelectric six-axis force sensor static calibration experimental system, which consists of a multi-axis force/torque loading device, an 8-channel quasi-static charge amplifier YE5850A, a signal pre-processing circuit for 8-channel to 6-channel conversion, a NI PCI-6259 high-speed data acquisition device, and the six-axis force sensor static calibration software based on LabView and MATLAB. In the static calibration experiment, the sensitivity test gear for the quasi-static charge amplifier was located at 1,000 Pc/unit. To ensure the accuracy of the static calibration, a parallel sampling method for the 6-channel input signal was carried out using the interval sampling principle. The experimental results indicate that the data transition rate of each channel is greater than 13 kHz and is able to meet the requirements of the static calibration experiment.

**Figure 6.**
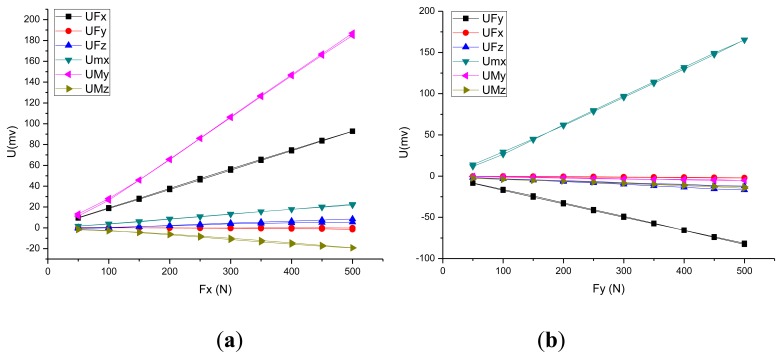
Selected input force and output voltage calibration curve of the sensor: (**a**) F_X_ direction, (**b**) F_Y_ direction.

Figure 6 shows F_X_ and F_Y_ direction input force and output voltage calibration curve for the piezoelectric six-axis force sensor. The curve shows that the experimental test results are consistent with the theoretical analysis results based on the analytical/mathematical model and the numerical simulation model:

(9) $$C_{Q_{m}}=\left[\begin{array}{cccccc} 2.016 & 0 & 0 & 0 & 2.899 & 0\\ 0 & 2.016 & 0 & -2.899 & 0 & 0\\ 0 & 0 & 2.306 & 0 & 0 & 0\\ 0 & 0 & 0 & 362.418 & 0 & 0\\ 0 & 0 & 0 & 0 & 362.418 & 0\\ 0 & 0 & 0 & 0 & 0 & 280.403 \end{array}\right]$$

(10) $$C_{Q_{s}}=\left[\begin{array}{cccccc} 2.052 & -0.003 & -0.002 & -0.006 & 2.778 & 0.000\\ -0.003 & 2.071 & -0.000 & -2.769 & 0.005 & -0.000\\ -0.000 & -0.000 & 2.237 & -0.000 & -0.001 & -0.000\\ -0.009 & -0.073 & -0.054 & 389.593 & 0.024 & -0.001\\ 0.095 & 0.020 & -0.065 & 0.116 & 389.540 & 0.000\\ 0.003 & -0.014 & 0.023 & 0.008 & 0.003 & 294.831 \end{array}\right]$$

(11) $$C_{Q_{e}}=\left[\begin{array}{cccccc} -1.869 & -0.001 & 0.097 & 0.416 & 3.321 & -0.342\\ -0.043 & 1.654 & -0.305 & 3.093 & -0.104 & -0.321\\ 0.047 & 0.096 & 2.877 & -0.329 & -0.275 & -0.067\\ -1.902 & -1.503 & -14.153 & 209.938 & 10.852 & -7.359\\ -5.494 & 4.003 & -14.547 & -29.676 & -212.685 & -15.088\\ 9.363 & -3.020 & 6.741 & -27.190 & -10.318 & 150.156 \end{array}\right]$$

According to the output charges *Q_ij_* of the piezoelectric six-axis force sensor, when the unit load (the force equivalent to 1N, the torque equivalent of 1 N·m) acts on it. The linear decoupling matrix *C_Q_* = [(*Q_ij_*)*^T^*]^−1^ of the piezoelectric six-axis force sensor can be constructed according to Equation (1).

In Equations (9–11), *C_Qm_* and *C_Qs_* are the linear decoupling matrices of the piezoelectric six-axis force sensor obtained via the analytical mathematical model and numerical simulation model, respectively, *C_Qe_* is the decoupling matrix obtained from the experimental prototype static calibration of the piezoelectric six-axis force sensor, and C_66_ are the elements of matrix *C_Qs_*, which is obtained from 4.4 times the linear correction.

Table 2 shows the static sensitivity of the piezoelectric six-axis force sensor obtained from the linear decoupling matrix *C_Qm_*, *C_Qs_* and *C_Qe_*. As listed in Table 2 and using the linear decoupling matrices *C_Qm_*, *C_Qs_* and *C_Qe_*, the following conclusions can be summarized:

(1) The sensitivities obtained from the analytical mathematical model and numerical simulation model are quite consistent; due to the influence of the equivalent methods, production precision and calibration accuracy, the sensitivity test results are lower than the design aim; due to the influence of charge amplifier's zero shift, in the *f_z_* direction, its sensitivity is higher than the design specifications.
(2) The cross-coupling between *f_x_*, *f_y_* to *m_y_*, *m_x_* obtained from the analytical mathematical model is 143.80%, and the remaining directions show no dimensional coupling. The cross-couplings between *f_x_*, *f_y_* to *m_y_*, *m_x_* obtained from the numerical model are 135.38% and 133.70%, and the other directions show no dimensional coupling. The cross-couplings between *f_x_*, *f_y_* to *m_y_*, *m_x_* obtained from the experimental prototype calibration are 177.69% and 187.00%; due to the influence of manufacturing precision, the highest level of cross coupling in the other direction reaches 18.11%,but these interferences are linear coupling, and can be eliminated using a mathematical compensation method.
(3) The force isotropy is 0.6531, 0.6467 and 0.7098, and the moment isotropy is 0.7737, 0.7567 and 0.6233, as obtained from the analytical mathematical model, numerical simulation model and experimental prototype of the piezoelectric six-axis force sensor, respectively. The force/torque isotropy of the analytical mathematical model is broadly consistent with that of the numerical simulation model. Because of the influence of machining accuracy, deviations are observed among the experimental prototype and the analytical mathematical model and numerical simulation model of the piezoelectric six-axis force sensor. However, due to the test gear, the quasi-static charge amplifier affects the sensitivity of the piezoelectric force sensor measurement system, and therefore, the force/torque isotropy of the piezoelectric six-axis force sensor could be greatly improved by selection of better test gear with respect to the charge amplifier.

## 5. Conclusions

In this paper, we have investigated a novel six axis force/torque sensor based on double quartz crystal chip groups. Its analytical mathematical model and numerical simulation model are presented. The research conclusions can be drawn as follows:

1. The sensor's static performances (*i.e.*, static sensitivity, impedance, anti-humidity) are better than six axis force/torque sensor based on signal quartz crystal chip groups ([3]).
2. Due to the influence of the six axis force/torque sensor's spatial structure, there are some cross coupling interferences take place in the *f_y_*, *m_x_*, *f_x_*, *m_y_* directions, and these static cross coupling interferences are linear coupling and can be eliminated using a mathematical compensation method.
3. These sensors' analytical mathematical model which is derived using the material mechanics and theoretical mechanics, and numerical simulation model based on ANSYS is presented, are effective. We can realize the design of these sensors' static performance through the two models.
4. Due to the influence of the simulation method, production precision and calibration accuracy, the test accuracy of the sensor experimental prototype in the *f_Z_* direction does not completely meet the design requirements, the simulation result in the *m_Z_* direction require linearity correction. These issues will be the focus of future research for implementing the static-active design of the piezoelectric multi-axis force senor based on a multi-point support structure.

## Figures and Tables

**Table 1.** Main structural parameters of six-axis force sensor model.

**Component**	**Thickness (mm)**	**Inner Diameter (mm)**	**Outside Diameter (mm)**	**Material**	**Elastic Modulus (Pa)**	**Density (Kg/m^3^)**
Cover	6	15	47	1Cr18Ni9Ti	2.1e11	7,900
Inner tube	12	15	16	1Cr18Ni9Ti	2.1e11	7,900
Shell	12	46	50	1Cr18Ni9Ti	2.1e11	7,900
Quartz crystal chip	1	-	10	SiO_2_	8.0e10	2,650

**Table 2.** Piezoelectric six-axis force/torque sensor sensitivity.

**Category**	**Sensitivity of Force (Pc/N)**			**Sensitivity of Torque (Pc/N.m)**		

	Sfx	Sfy	Sfz	Smx	Smy	Smz
Analytical model	2.016	2.016	2.306	362.418	362.418	280.403
Numerical model	2.052	2.071	2.237	389.593	389.540	294.831
Experimental prototype	1.869	1.654	2.877	209.938	212.685	150.156
